# Mature (Benign) Cystic Retroperitoneal Teratoma Involving the Left Adrenal Gland in a 22-Year-Old Male: A Case Report and Literature Review

**DOI:** 10.1155/2013/610280

**Published:** 2013-05-16

**Authors:** Adnan Bhatti, Hindi Al-Hindi, Ayman Azzam, Tarek Amin, Ahmed Abu-Zaid

**Affiliations:** ^1^Department of Surgical Oncology, King Faisal Specialist Hospital and Research Center (KFSH&RC), P.O. Box 3354, Riyadh 11211, Saudi Arabia; ^2^Department of Pathology and Laboratory Medicine, King Faisal Specialist Hospital and Research Center (KFSH&RC), P.O. Box 3354, Riyadh 11211, Saudi Arabia; ^3^Department of General Surgery, Faculty of Medicine, Alexandria University, Alexandria 21526, Egypt; ^4^College of Medicine, Alfaisal University, P.O. Box 50927, Riyadh 11533, Saudi Arabia

## Abstract

Primary retroperitoneal teratomas involving adrenal glands are exceedingly uncommon accounting for only 4% of all primary teratomas. They are more common in childhood and rarely occur in adults. Only a very few case reports have been documented in literature so far. Herein, we report a mature (benign) cystic retroperitoneal teratoma in the region of left adrenal gland in a 22-year-old otherwise healthy male patient who presented with a 1-month history of left flank pain. In addition, a literature review on teratomas is included.

## 1. Introduction

Primary mature teratomas are uncommon nonseminomatous germ cell tumors. They are made up of well-differentiated parenchymal tissues that are derived from more than one of the three germ cell layers (ectoderm, mesoderm, and endoderm) [1]. They usually occur in midline (paraxial) structures. The most common sites are gonads (testes and ovaries) followed by extragonadal sites such as intracranial, cervical, mediastinal, retroperitoneal, and sacrococcygeal regions [2]. Primary retroperitoneal teratomas involving adrenal glands are exceedingly uncommon accounting for only 4% of all primary teratomas [2–4]. They are more common in childhood and rarely occur in adults [5]. Only a very few case reports have been documented in literature so far [6]. The majority of cases are asymptomatic, present with nonspecific complaints, or identified incidentally on routine investigations [7]. Surgical excision of mature (benign) teratoma is required for a definitive diagnosis (by histopathological examination) [8] and remains the mainstay of treatment [9]. Prognosis is fortunately excellent after complete surgical excision with an overall five-year survival rate of nearly 100% [10]. Herein, we report a mature (benign) cystic retroperitoneal teratoma in the region of left adrenal gland in a 22-year-old otherwise healthy male patient who presented with a 1-month history of left flank pain.

## 2. Case Report

**Figure 1:**
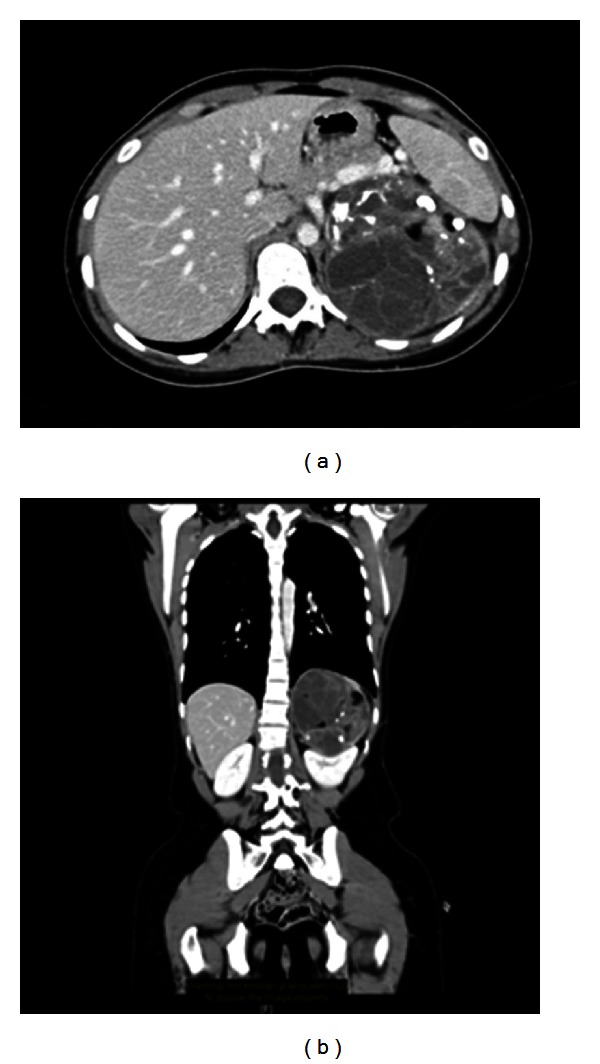
Abdominal contrast-enhanced computed tomography (CT) scan. (a) Cross-sectional (transverse) view: showing a large lesion in the region of left adrenal gland measuring 9.0 × 9.2 × 10.8 cm and demonstrating multiple cystic spaces with thin septations as well as multiple areas of fatty collections and coarse calcifications. (b) Coronal (frontal) view: showing displacement of left kidney inferiorly.

A 22-year-old otherwise healthy male patient presented to King Faisal Specialist Hospital and Research Centre with a 1-month history of left flank pain. Physical examination was remarkable for a palpable, nontender, and limitedly mobile mass on the left flank. All laboratory investigations were unremarkable. An abdominal contrast-enhanced computed tomography (CT) scan showed a large lesion in the region of left adrenal gland measuring 9.0 × 9.2 × 10.8 cm and demonstrating multiple cystic spaces with thin septations as well as multiple areas of fatty collections and coarse calcifications (Figure 1(a)). The huge space-occupying lesion displaced the left kidney inferiorly (Figure 1(b)). Bearing in mind a potential malignant tumor mass in the retroperitoneum (i.e., liposarcoma), the surgical oncology team advised for a surgical resection.

**Figure 2:**
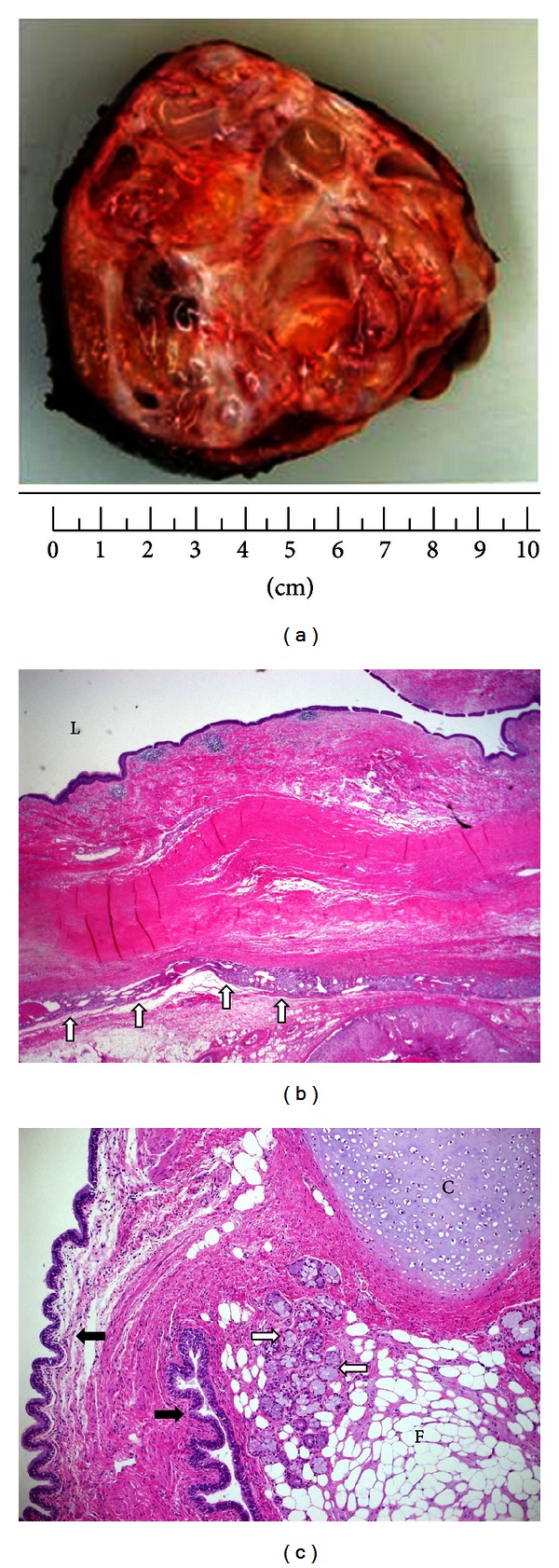
Mature (benign) cystic retroperitoneal teratoma in the region of left adrenal gland. (a) Grossly, cut-section of the resected mass revealed multilocular cystic spaces, whitish-gray walls, scattered yellowish adipose tissues, mucus secretions, and areas of calcifications. (b) Hematoxylin & Eosin (H&E stain), ×20 magnification: a scanning magnification view of the lesion depicting its relationship with the adrenal cortex (open arrows). The lumen [L] is lined by respiratory columnar epithelium. (c) Hematoxylin & Eosin (H&E stain), ×40 magnification: a low magnification image of another cystic space lined by mucous-secreting epithelium (solid arrows). The wall is formed by cartilage tissues [C], fat (adipose) tissues [F], and salivary gland tissues (open arrows).

The patient underwent complete surgical resection of the left adrenal mass. Grossly, the left adrenal mass weighted 484 g and measured 9.0 × 9.0 × 10 cm. The mass had well-circumscribed smooth borders and rubbery consistency. Cut-section of the mass revealed multilocular cystic spaces, whitish-gray walls, scattered yellowish adipose tissue collections, mucus secretions, and areas of calcifications (Figure 2(a)). Microscopically, the walls were largely lined by respiratory columnar and squamous epithelium with various proportions of mature well-differentiated parenchymal tissues derived from the various three germ cell layers (Figures 2(b) and 2(c)). No evidence of malignancy was identified. A diagnosis of mature (benign) cystic retroperitoneal teratoma involving the left adrenal gland was made.

Since there was no evidence of immature or malignant components, no radio- or chemotherapy was offered. The patient was discharged uneventfully in a stable condition. A postoperative 6-month followup failed to show any evidence of tumor recurrence.

## 3. Discussion

Germ cell tumors (GCTs) can be broadly classified into two main categories: seminomatous and nonseminomatous GCTs. Teratomas belong to nonseminomatous GCTs and represent the most common form of all GCTs [8]. Teratomas are encapsulated neoplasms composed of multiple parenchymal tissues (of varying degrees of differentiation) that are derived from more than one germ cell layer (ectoderm, mesoderm, and endoderm) [3].

Generally, teratomas arise from uncontrolled proliferation of pluripotent cells: germ cells and embryonal cells. The type of pluripotent cell greatly influences the presentation time and involved location of teratoma. Teratomas of germ cell sources can be congenital or acquired and are usually found in gonads (testes and ovaries). In contrast, teratomas of embryonic cell sources are always congenital and are usually found in extragonadal locations, such as intracranial, cervical, retroperitoneal, mediastinal, and sacrococcygeal sites [2, 4].

According to the location of tumor, teratomas can be classified into gonadal and extragonadal teratomas. Gonadal teratomas are more common, mostly primary neoplasms, mainly in adults, and usually take place in gonads (testes and ovaries) [7]. Conversely, extragonadal teratomas are less common, mostly secondary neoplasms, mainly in infants and young children [7], and usually take place in sacrococcygeal, mediastinal, retroperitoneal, and pineal gland sites (descending order of frequency) [11–13].

Furthermore, according to the content of tumor, teratomas can be classified into solid, cystic, or mixed teratomas. Solid teratomas lack organization and contain only parenchymal tissues. Cystic teratomas contain only sacs of fluid, semifluid, or fat, whereas mixed teratomas contain both solid and cystic components [4].

Besides, according to the epithelial lining and dermal contents of tumor, teratomas can be classified into epidermoid, dermoid, and teratoid teratomas (cysts). Epidermoid teratomas are lined by stratified squamous epithelium and lack dermal contents. Dermoid teratomas are mostly lined by stratified squamous epithelium and contain various dermal contents such as hair, sweat, and sebaceous glands. Teratoid teratomas are mostly lined by respiratory columnar epithelium and contain sebum [4].

In addition, according to the degree of tumor maturation, teratomas can be classified into mature and immature teratomas. Mature teratomas are generally benign, asymptomatic and more common, among females. They are highly variable on histology and can be solid, cystic, or mixed. They contain different types of parenchymal tissues that are well differentiated. Mature cystic teratomas (AKA dermoid cysts) may have partially to completely well-developed organ systems. On the contrary, immature teratomas are histologically solid teratomas and contain immature (undifferentiated/undeveloped) parenchymal tissues and can be possibly benign, possibly malignant, or frankly malignant. They are more common among males [8, 14].

Some mature (benign) and immature (possibly benign or possibly malignant) teratomas have an increased tendency to become frankly malignant teratomas, and frankly malignant teratomas have an increased propensity to metastasize. This group of exceptionally rare teratomas is known as teratomas with malignant transformation [8]. The stratified squamous epithelial components of these teratomas are the ones at an increased risk of undergoing malignant transformations. In addition, teratomas with malignant transformation may produce components of somatic (non-germ cell) neoplasms such as carcinoma, sarcoma, and leukemia [15, 16].

Occasionally, a teratoma may contain various components of other germ cell tumor, and hence it is not a pure teratoma per se, but rather it is a mixed germ cell tumor and has malignant nature. In infants and young children, these components are frequently endodermal sinus tumor and choriocarcinoma. A pure teratoma can be benign, however, highly aggressive in its clinical course as in a growing teratoma syndrome (GTS). GTS refers to a rapidly growing pure mature (benign) teratoma that appears during or following chemotherapeutic eradication of malignant components of a nonseminomatous germ cell tumor, and it has normal serum tumor marker levels of alpha-fetoprotein and human chorionic gonadotropin [17].

The vast majority of retroperitoneal teratomas are secondary neoplasms and mostly occur in males [15]. Primary retroperitoneal teratomas are extremely unusual neoplasms accounting for approximately 1–11% of all primary retroperitoneal neoplasms and typically occur in neonates, infants, and children age groups [13]. In adults, these neoplasms commonly present in the third or fourth decades of lives [18]. 

Primary retroperitoneal teratomas involving adrenal glands are exceedingly uncommon accounting for only 4% of all primary teratomas [2–4] and can be mistaken for other histologically related lipomatous adrenal neoplasms [7]. They are more common in childhood and rarely occur in adults [5].

Only a few case reports have been documented in literature so far [6]. They are more frequently encountered at the left side [3, 4]. The majority of cases are asymptomatic, present with nonspecific complaints, or identified incidentally on routine investigations [7].

Teratomas can be diagnosed based on high index of clinical suspicion, routine laboratory, and radiographic investigations [18]. With respect to high index of clinical suspicion, retroperitoneal teratomas involving adrenal glands may present congenitally, or later in life when they grow to massive sizes [19]. Clinical presentations are variable and include nonspecific, abdominal/flank/back pain, obstructive gastrointestinal and genitourinary symptoms, as well as lower limb/genital swelling due to lymphatic obstruction [18]. They can rarely present with complications such as secondary infections (abscess formation) [20], traumatic rupture leading to acute peritonitis [21], or malignant transformations [22]. Midline (paraxial) teratoma masses, with restricted mobility, can be easily detected on physical examination [23]. 

With respect to laboratory investigations, retroperitoneal teratomas can express a diversity of serum tumor markers such as elevated alpha-fetoprotein (AFP), carcinoembryonic antigen (CEA), and CA 19-9 [11–13]. These serum tumor markers are helpful in clinical practice and can be used to monitor successful treatment or detect relapse in patients with specific tumor marker-secreting teratomas. 

With respect to radiographic investigations, they play valuable roles in diagnosis of teratomas. Plain radiographs (X-ray) can identify calcified elements in 62% of cases [11–13] whereas ultrasound (US) can greatly differentiate between cystic and solid elements [18]. Computed tomography (CT) scans can better distinguish between fat (adipose tissue) and bone (calcified) masses [24]. On the contrary, magnetic resonance imaging (MRI) scans can offer better resolution of soft tissues, feasible identification of benign and malignant neoplastic features, and most importantly superior tumor staging assessment [25]. However, generally, a definitive diagnosis of teratoma demands a histopathological evaluation [8].

Surgical excision of benign (mature) teratoma is required for a definitive diagnosis (by histopathological examination) [8] and remains the mainstay of treatment [9]. Prognosis is fortunately excellent after complete surgical excision with an overall five-year survival rate of nearly 100% [10]. Teratomas are largely resistant to radio- and chemotherapy. Adjuvant radio- and chemotherapy are used only if malignant features of germ cell tumors are identified on histopathological examination [19]. A testicular ultrasound (US) is highly advised to rule out potential coexisting germ cell tumors (GCTs) as approximately 50% of men with retroperitoneal teratomas have testicular carcinomas in situ at the time of diagnosis, which, if left untreated, can develop into testicular germ cell tumor [26].

## 4. Conclusion

Primary retroperitoneal teratoma involving the region of adrenal gland is exceedingly rare (4% of all primary teratomas), and its occurrence in an adult is exceptionally uncommon. However, it should be regarded in the differential diagnosis in any patient presenting with a flank pain. Histopathological examination of the resected tumor warrants a definitive diagnosis. Surgical excision of mature (benign) teratoma remains the mainstay of treatment with an excellent five-year survival rate of nearly 100%.
